# A fragile effect: The influence of episodic memory on delay discounting

**DOI:** 10.1177/17470218241239289

**Published:** 2024-03-31

**Authors:** Nicky Duff, Rebecca Olsen, Zoe Walsh, Karen Salmon, Maree Hunt, Anne Macaskill

**Affiliations:** School of Psychology, Victoria University of Wellington, Wellington, New Zealand

**Keywords:** Delay discounting, episodic memory, episodic future thinking, semantic future thinking, order effects

## Abstract

Delay discounting occurs when a reward loses value as a function of delay. Episodic future thinking (EFT) reliably decreases delay discounting. EFT may share cognitive features with recalling episodic memories such as constructive episodic simulation. We therefore explored whether recalling episodic memories also reduces delay discounting. In Experiment 1, participants wrote about episodic memories and recalled those memories before completing a delay discounting task. Episodic memories reduced delay discounting according to one commonly used delay discounting measure (area under the curve) but not another (using the hyperbolic model). Experiment 2 compared the effects of general and episodic memories. Neither general nor episodic memories significantly decreased delay discounting compared with a control “counting” condition, but episodic memories reduced delay discounting compared with general memories under some conditions. In Experiment 3, episodic memories did not decrease delay discounting compared with three other control conditions while EFT did. Experiment 3 therefore found that thinking must be both episodic *and* future orientated to reduce delay discounting. Together, these results suggest that episodic thinking is not sufficient to reliably decrease delay discounting, rather, features unique to episodic *future* thinking are required. Episodic memory might reduce delay discounting in some contexts, but this effect is small and fragile.

Delay discounting refers to the fact that rewards lose value as a function of delay (Mazur, 2015). As a result, in day-to-day life people might choose smaller, sooner rewards (e.g., spending on frivolous items now) over larger later rewards (e.g., saving for a bigger, more important item later). The most common laboratory measure of delay discounting is to ask participants to make hypothetical choices between a smaller sum of money now and a larger sum of money later (e.g., Critchfield & Kollins, 2001). Choosing the larger sum more often indicates lower levels of delay discounting (i.e., shallower discounting) which is sometimes referred to as self-control. Conversely, choosing the smaller, sooner indicates higher levels of delay discounting (i.e., steeper discounting), which is sometimes referred to as impulsivity. Higher levels of delay discounting can impact health and wellbeing (Critchfield & Kollins, 2001; Reynolds, 2006), and are associated with substance dependence (Bickel & Marsch, 2001; Dallery & Raiff, 2007), obesity (Epstein et al., 2010), and problem gambling (Petry, 2001).

Episodic future thinking (EFT) is one strategy to help people pick larger, later options (Rung & Madden, 2018). When people are cued by an experimenter to imagine possible positive, future events, they are more likely to choose the larger, later sum of money (Liu et al., 2013; Rung & Madden, 2018). EFT decreases delay discounting in adults and children (Daniel et al., 2015), and can also help to reduce cigarette consumption (Stein et al., 2016), and food intake (Daniel et al., 2013). As there is a well-established effect of EFT on delay discounting (Rung & Madden, 2018), it is reasonable to examine whether other forms of episodic thinking may also have an effect. The question therefore arises whether episodic memory (i.e., memory of past experiences; Tulving, 2002) can also decrease delay discounting.

Some EFT studies have used episodic memory as a control and have found that EFT was significantly better at decreasing delay discounting than episodic memory (Dassen et al., 2016; O’Donnell et al., 2017; Stein et al., 2016). This does not rule out the possibility that episodic memory enhances self-control compared with a non-episodic-thinking control condition, however. Indeed, the effect of EFT on delay discounting may depend on several factors, like the future orientation, valence, and episodic nature of the thinking. Researchers have therefore begun to explore which components of EFT are necessary to decrease delay discounting (Rung & Madden, 2018). One of the components worth exploring is the orientation of the thinking. That is, if thinking of a future, positive event decreases delay discounting, could changing the orientation of the imaging to a *past*, positive event—an episodic memory—also be helpful, even if to a smaller degree?

Theoretical and empirical evidence suggests that episodic memory may help to decrease delay discounting. For instance, the “constructive episodic simulation hypothesis” posits that future and past episodic thinking activate similar cognitive and neural processes in the brain (Addis, 2018; Schacter & Addis, 2007). That is, both episodic past and future thinking require constructing mental events in the mind. This cognitive process of assembling and maintaining a coherent mental scene has been termed “event construction” (Romero & Moscovitch, 2012; Schacter & Madore, 2016), “scene construction” (Hassabis & Maguire, 2007), or “simulation” (Addis, 2018). Thus, if we are using the same cognitive and neural processes to recall the past and imagine the future (i.e., generate a mental scene), then episodic memory might reduce delay discounting as EFT does.

Lempert and colleagues (2017) provided experimental evidence for positive episodic memories decreasing delay discounting. Their study was a within-participant two-part study. In the first session, participants recalled positive episodic memories. In the second session, 3 days later, they were asked to recall the most positively rated memories from the first session before completing the delay discounting task. For the control condition, participants were asked to relax. Participants had significantly lower levels of delay discounting after recalling episodic memories compared with the control, with a medium effect size (Cohen’s *d* of 0.4). The effect was replicated in a follow-up study, with a slightly smaller effect size (Cohen’s *d* of 0.29). Lempert and colleagues concluded that the temporal focus of the mental imagery does not necessarily have to be future orientated to reduce delay discounting. They also posited that perhaps recalling positive episodic memories evokes nostalgia which may help to reduce delay discounting. Heightened nostalgia has been shown to make people more future orientated (Sedikides et al., 2016), and feeling connected to one’s future self has been linked to lower levels of delay discounting (Hershfield et al., 2011; Lempert et al., 2017). Furthermore, Ciaramelli and colleagues (2019) found that participants who recalled vivid episodic memories had lower delay discounting rates than people who attended to their present experience. Ciaramelli and colleagues concluded that self-projection away from the present may dampen the appraisal of an immediate reward (also see Smallwood et al., 2013).

Thus, the research summarised above suggests that episodic memory may also reduce delay discounting (i.e., Ciaramelli et al., 2019; Lempert et al., 2017; Schacter & Addis, 2007), perhaps in a similar way that EFT may help to decrease delay discounting: by using the same cognitive processes as future thinking (Addis, 2018; Schacter & Addis, 2007), connecting to one’s future self (Hershfield et al., 2011; Lempert et al., 2017), and diverting attention away from the present (Ciaramelli et al., 2019; Smallwood et al., 2013). Therefore, it is worthwhile investigating whether episodic memory reduces delay discounting further.

## Research aim: experimentally test whether episodic memory decreases delay discounting

To date, the studies outlined above are the only published experimental research that has found an effect of episodic memories on delay discounting, with one other study finding *no* effect in older adults (Lempert et al., 2020). Perhaps, therefore, the effect of episodic memories on delay discounting is fragile. That is, perhaps the effect of episodic memories is small and depends on factors like how the episodic memory is recalled and the method used to assess delay discounting. Thus, here we present three experimental studies that investigated whether positive episodic memory decreased delay discounting under different experimental conditions. The preregistration for Experiment 1, and the derived, constructed data and supplementary material (e.g., task instructions and memory cues) for all the experiments outlined in this article can be found on Open Science Framework (https://osf.io/62gxb/?view_only=)^1^ The university’s Human Ethics Committee approved of all three experiments (#0000024179).

## Experiment 1: Do episodic memories decrease delay discounting?

Experiment 1 aimed to replicate Lempert and colleagues’ (2017) study that found positive episodic memories decreased delay discounting. Ciaramelli et al. (2019) also offered a possible procedure to replicate. Lempert and colleagues observed an effect of episodic memory on delay discounting in their primary analysis, while Ciaramelli et al. only observed it in a post hoc analysis. We therefore selected the Lempert et al. procedure because it offered a better starting point to determine whether the effect of episodic memory on delay discounting is replicable, as well as identify the conditions under which the effect occurs. Considering Lempert and colleagues’ findings, we hypothesised that participants would have lower delay discounting rates in the episodic-memory condition compared with the control (i.e., relax) condition.

### Method

#### Participants

A sample size of 72 participants was preregistered. G*Power (Faul et al., 2007) estimated that 54 participants were needed to test for a medium effect size (like in Lempert et al., 2017), with a two-tailed paired-sample *t*-test, an alpha set to .05, and power to .95. We registered and collected more participants to account for unsystematic data, however (see below for more details; Johnson & Bickel, 2008; K. R. Smith et al., 2018).

A total of 71 undergraduate students from Victoria University of Wellington participated in partial fulfilment of a course requirement. We did not collect any additional demographic information. Of them, 3 completed the task in the second session incorrectly, and 13 had unsystematic data in the control condition (see below for more information); therefore, the final sample consisted of 58 participants.

#### Procedure

This was a within-participant, two-part experiment (i.e., two sessions, 3 days apart) that followed Lempert and colleagues’ (2017) experimental design as much as possible. Changes to the Lempert et al. design are noted below. Each session took approximately 45 min.

##### Session 1

At the start of Session 1, participants read information about the experiment and were asked to complete consent procedures. Participants then picked 10 memory cues from a list (e.g., a time I hosted a party/a time I went to the beach; adapted from Lempert et al., 2017). Participants recalled a positive, episodic memory that corresponded to each cue, wrote a description of each, and wrote their own memory cue which helped them to recall their memory in the second session. Participants were told that memories should be positive experiences. If they could not recall a positive memory, however, they were asked to select a neutral memory, but not a negative one. For each memory, on a 4-point scale, participants rated valence (i.e., *how positive is this memory?* 1 = *neutral*, 4 = *very positive*), emotional intensity (i.e., *how intense is this memory?* 1 = *not intense*, 4 = *very intense*), and feeling (i.e., *how good does this memory make you feel?* 1 = *neutral*, 4 = *very good*). Furthermore, to ensure that their memories were episodic (i.e., specific experiences), participants were asked to type in the approximate location and date of their experience. Participants had 2 min to type each memory and were encouraged to write as much as possible within that timeframe. After 2 min, a button (“next memory”) appeared, and the participant clicked it to move on to writing about the next memory.

##### Session 2

Participants returned 3 days later and participated in an episodic-memory condition and a control condition. The second session had four blocks: two episodic-memory blocks and two control (i.e., relax) blocks. An episodic-memory block had five episodic-memory cues and a control block had five “relax” cues. Half of the participants started with an episodic-memory block, and the other half started with the control block. Episodic memory and control blocks then alternated. For each cue, participants made six delay discounting choices. Thus, each participant completed 120 delay discounting choices (i.e., 60 choices per condition).

**Figure 1.**
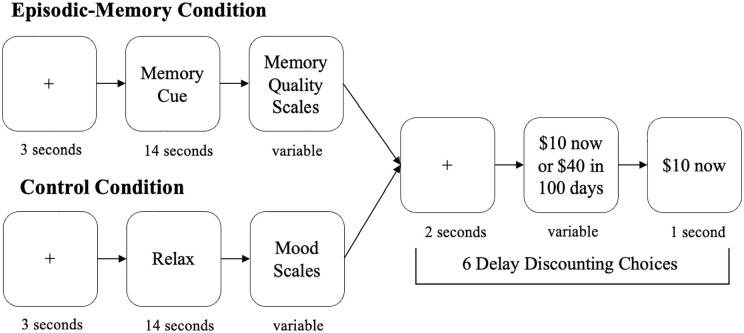
Session 2, Experiment 1: Episodic memory and control conditions. *Note.* Figure adapted from Lempert et al. (2017). This figure shows the sequence of events in each condition: thinking about a cue, completing scales, and making delay discounting choices. See text for more information.

For the episodic-memory condition, a block began with a fixation cross on the screen for 3s, followed by 14 s of exposure to a memory cue they had written in the first session (see Figure 1). Participants were asked to recall and think about the memory that corresponded to their memory cue. Memory cues were presented at random and did not correspond to the order in which they were written in the first session. Next, participants filled in the same scales assessing valence, intensity, and feeling as in the first session. In the control condition, participants were asked to relax and rate how bored (1 = *not bored*, 4 = *very bored*), tired (1 = *very awake*, 4 = *very tired*), and good (1 = *neither good nor bad*, 4 = *very good*) they felt.

After completing the scales, participants completed six delay discounting choices, in which they chose between a smaller sum of money now or a larger sum of money later. Choice options were determined identically for control and episodic-memory blocks as follows: the delays were 4, 7, 30, 60, 100, and 180 days, and the amounts were NZD$11, NZD$15, NZD$18, NZD$20, NZD$22, NZD$25, NZD$28, NZD$32, NZD$35, NZD$40; with “NZD$10 now” always being the fixed, smaller sooner option (following Lempert et al., 2017). The options switched sides of the screen randomly and participants made decisions about every possible combination of delay and amount in random order. That is, delay amount combinations were assigned at random to each block. Rewards were hypothetical, but research suggests that delay discounting is similar for real and hypothetical rewards (see Lagorio & Madden, 2005)

#### Data analysis

##### Estimating the indifference points

The first step to estimating each participant’s delay discounting rate was to identify the impact of the delay on the reward value. That is, for each participant we determined the indifference point at each delay: how large the reward had to be at a particular delay to be worth the same as NZD$10 now. Therefore, following Lempert and colleagues (2017; also see Kable & Glimcher, 2007), we used a logistic function (Equation 1):

(1) $$f\left(x\right)=\frac{L}{1+e^{-\beta (x-x_{0})}}$$

*L* is the curve’s maximum value (here, set to 1); *e* is natural logarithm base (i.e., 2.71828); β is the logistic growth rate (i.e., steepness of the curve, note Lempert et al. used *k* to refer to this parameter) and is a free parameter; and $x_{0}$ is the sigmoid point, a free parameter, and is the point where the probability of the participant picking the smaller, sooner amount is equal (0.5) to the probability of them picking the larger, later option at a particular delay (i.e., the indifference point). We used the Solver function in Excel to identify the value of β (starting value 10) and $x_{0}$ (starting value NZD$23) that minimised the sum of squared errors (Reed et al., 2012). The slope was also constrained to be greater than or equal to .1. The sample’s median *R*^2^ value for the indifference points for both conditions (*Mdn* = .79, *IQR* = .38 – 1.0) indicated good model fit.

Next, for each delay, NZD$10 (the smaller, sooner amount) was divided into the indifference point (i.e., the sigmoid point) to obtain the fraction of the objective value that the larger, later option retained at that delay. Indifference points could therefore range between 0 (the reward has no value at that delay) and 1 (the reward has the same value if received at that delay to that if received immediately).

##### Estimating the delay discounting rate

After estimating the subjective value of the delayed reward at each delay, the next step was to calculate the delay discounting rate. The delay discounting rate summarises the effect that delaying the reward has on the reward’s value for that individual and serves as the key dependent variable analysed. We used the hyperbolic model (Equation 2; Lempert et al., 2017; Mazur, 1987) and the area under the curve (AUC; Equation 3; Myerson et al., 2001) to identify the delay discounting rate.

Hyperbolic model (Equation 2).

The equation for the hyperbolic model is:

(2) $$V=\frac{A}{\left(1+kD\right)}$$

In Equation 2, *V* is the subjective value of the delayed reward which was estimated using Equation 1. *A* is the undiscounted value of the delay reward (i.e., 1), *D* is the delay manipulated by the experimenter across trials. *k* is a free parameter indicating the slope of the (hyperbolic) relationship between delay and subjective value and is also called the delay discounting rate. Using the Excel solver function, least squares regression was used to identify *k* for each participant in each condition. The median *R*^2^ value for *k* for the control condition (*Mdn* = .78; *IQR* = .40 − .89) and episodic-memory condition (*Mdn* = .61; *IQR* = .24 − .84) indicated relatively good model fit. For the delay discounting rate parameter *k*, *smaller* numbers indicate lower levels of delay discounting.

AUC (Equation 3).

Although Lempert et al. (2017) did not use AUC, AUC was also calculated because results can differ depending on whether *k* or AUC is adopted (C. L. Smith & Hantula, 2008; Ye et al., 2022). Estimating both delay discounting rates can be useful to identify situations in which the pattern of results depends on specific modelling assumptions researchers make. AUC summarises the area under the delay discounting curve. This is the curve with delay to receipt of the reinforcer on the *x-*axis and the subjective value of the reinforcer on the *y-*axis. Thus, the following equation was used to identify AUC:

(3) $$\sum^{\;}(X_{2}-X_{1})\frac{Y_{1}+Y_{2}}{2}$$

In Equation 3, *X* values are subsequent delay lengths and *Y* values are subsequent indifference points. AUC does not assume that the discounting function takes any particular form, while the hyperbolic model assumes a hyperbolic form (Myerson et al., 2001). AUC therefore indicates the proportion of the total possible area of the graph that is under the observed delay discounting curve. Thus, AUC ranges from 0 to 1, with *larger* numbers indicating lower levels of delay discounting.

##### Effect size

The following Equation 4 (Rosenthal, 1994) was used to calculate the effect size:

(4) $$r=\frac{z}{\sqrt{N}}$$

where *r* is the effect size, *z* is the *z*-score or the standardised test statistic, and *N* is the sample size.

##### Unsystematic discounting as a function of delay

The data of participants from the control condition who had unsystematic indifference points were removed from the analysis (i.e., listwise exclusion). Unsystematic indifference points were defined as not declining monotonically as a function of delay (Johnson & Bickel, 2008) and we considered that this indicated a lack of attention or understanding in the control condition. Johnson and Bickel (2008) set out two criteria for identifying non-systematic discounting data: (1) if any indifference point is greater than the indifference point at the preceding delay by more than 20% of the larger, later reward; and (2) if the last indifference point is not less than the first indifference point by at least 10% of the larger, later reward. Thirteen participants (18%) demonstrated unsystematic discounting in the control condition and their data from both the episodic memory and control conditions were therefore removed from the analysis (i.e., listwise exclusion; see Table 1). This rate of exclusion was typical for student populations (see meta-analysis by K. R. Smith et al., 2018).

**Table 1.** Experiment 1: Number of participants who met exclusion criteria in the control and episodic-memory conditions.

Control condition only (excluded)	Episodic-memory condition only (retained)	Both conditions (excluded)
4 (6%)	4 (6%)	9 (13%)

The aim of excluding participant data based on Johnson and Bickel’s (2008) criteria was to improve data quality, which is a common approach in the delay discounting research (K. R. Smith et al., 2018). We removed participants demonstrating unsystematic discounting in the control condition from all analyses. Thus, we did not exclude the four participants with unsystematic discounting in the episodic-memory condition but systematic discounting in the control condition. Such participants demonstrated attention and understanding of the task through their systematic discounting in the control condition. Their unsystematic discounting in the episodic-memory condition might reflect the effect of the episodic memory cues themselves; for example, a participant for whom recalling memories markedly reduced delay discounting might show very shallow discounting that “failed” the second criterion above.

There are multiple possible exclusion strategies, all of which have advantages and disadvantages (e.g., balancing increased sample size through retaining participants with improved data quality through excluding potentially inattentive participants). A disadvantage of the approach we took was the risk of creating an unattended confound, whereby a significant effect is due to exclusions rather than the independent variable (i.e., Type 1 error). To mitigate this risk, all our experiments are within-participant design, and we removed participant data from both conditions if they met the exclusion criteria in the control condition (i.e., listwise exclusion). In addition, wherever we observed a significant effect we tested whether this effect still occurred when all participants’ data were retained (see supplementary material). We found that significant effects reported reflect the experimental manipulation and not the exclusion criteria and thus no evidence that our exclusion strategy produced any such Type 1 errors.

### Results and discussion

#### Descriptive statistics, normality testing, and statistical analyses plan

Using the Shapiro–Wilk test (and visual inspection of histograms depicting data distributions), AUC and *k* scores for the episodic-memory and control conditions were tested for normality: AUC episodic-memory condition (*W* = .88, *p* < .001), AUC control condition (*W* = .85, *p* < .001), *k* episodic-memory condition (*W* = .70, *p* < .001), and *k* control condition (*W* = .74, *p* < .001) scores had non-normal distributions. Thus, the median and IQR were calculated for AUC and *k* scores (see Table 2) and non-parametric tests were used when analysing differences between conditions (as outlined in our preregistration). This approach for managing non-normal distributions differed from Lempert and colleagues’ (2017) study, which is discussed further below. Note that all *p*-values reported in this article are two-tailed.

**Table 2.** Experiment 1: Descriptive statistics.

			Second session			
	First session		Episodic-memory condition		Control condition	
Measure	Median	IQR	Median	IQR	Median	IQR
AUC	–	–	.38	.33–.50	.35	.31–.48
*k*	–	–	.03	.02–.05	.03	.02–.05
Valence (i.e., positivity)	3.3	3.1–3.5	3.4	2.9–3.6	–	–
Intensity	2.5	2.0–2.9	2.5	2.1–3.1	–	–
Feeling	3.4	3.2–3.7	3.5	3.2–3.8	–	–
Bored	–	–	–	–	3.0	2.3–3.4
Tired	–	–	–	–	3.0	2.5–3.5
Good	–	–	–	–	2.0	1.5–2.5

*Note. N* = 58. A lower *k* and a higher AUC indicate lower levels of delay discounting, whereas a higher *k* and lower AUC indicate higher levels of delay discounting. Scales in the bottom six rows of the table ranged from 1 to 4, with higher scores indicating higher levels of that factor.

The sample on average rated their memories positively and indicated that they produced positive feelings, and that these scores were relatively stable across the two sessions. The positivity scale’s IQR for both sessions were narrow, which indicates that participants followed instructions and wrote positive memories.

#### Key finding: positive episodic memories decreased delay discounting

**Figure 2.**
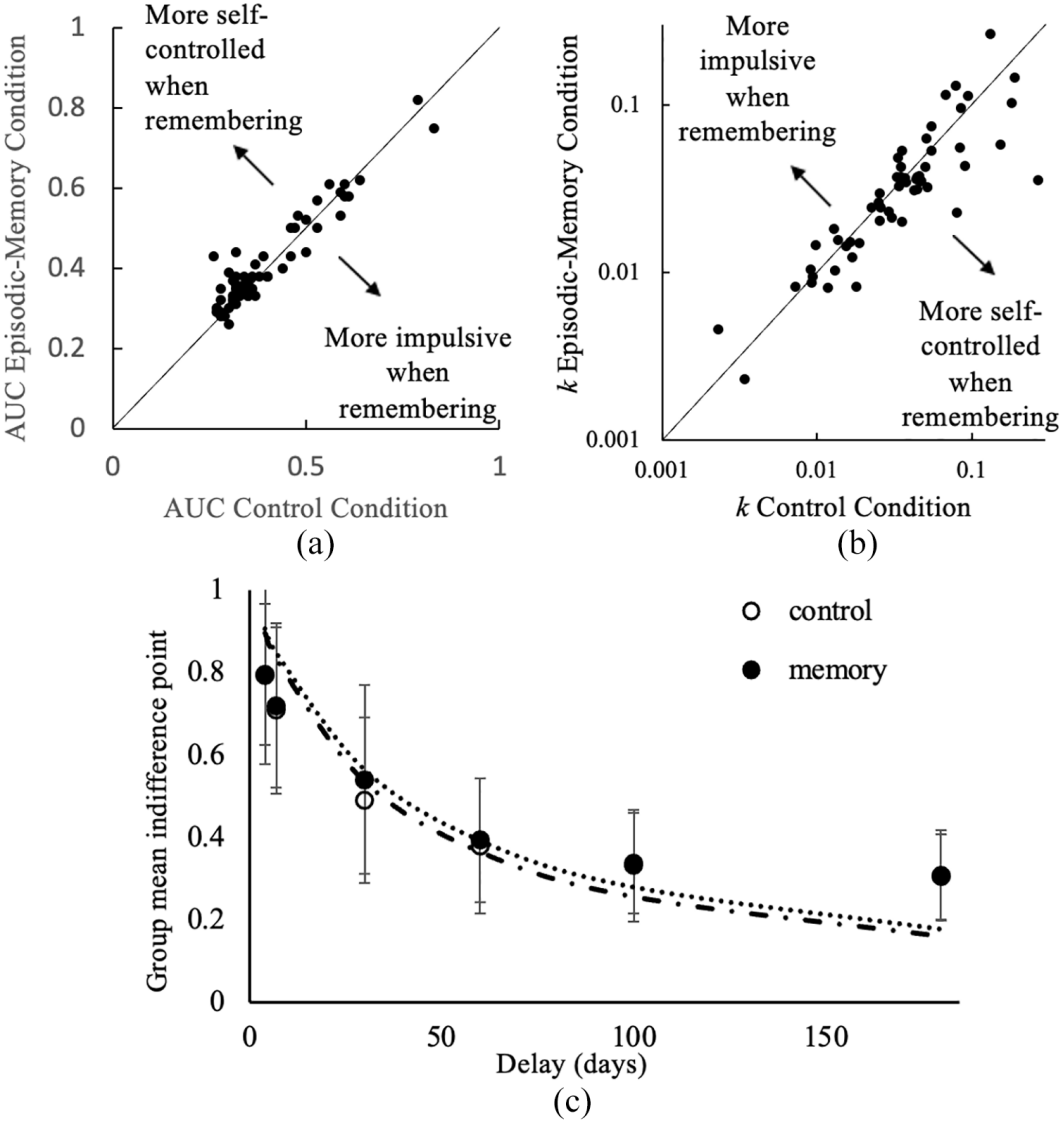
Delay discounting in control and memory conditions. *Note.* Modified Brinley plots that show the relationship between episodic-memory condition and control condition for Experiment 1. Data points indicate participants’ (a) AUC and (b) *k* scores. Data points on the line show that both conditions yielded the same scores for both conditions, whereas data points above or below the line indicate a change in delay discounting rates between conditions. Note logged axes on (b). (c) Graph shows group mean delay discounting curves. Error bars depict standard error of the mean. Dashed lines are best fitting hyperbolic functions.

For the AUC measure, 35 of 55 participants (63%) demonstrated lower levels of delay discounting in the episodic-memory condition (see points above the reference line, left graph, Figure 2), which suggests that episodic memories decreased delay discounting. Consistent with the hypothesis, a Wilcoxon matched pair signed-rank test indicated that participants discounted significantly less in the episodic-memory condition compared with the control condition, *z* = −2.03, *p* = .042. The effect size (using AUC) was *r* = .21, indicating a small-to-medium effect size (Field, 2017). In contrast, when *k* was used to characterise delay discounting, although 32 of 55 participants (58%) demonstrated lower levels of delay discounting in the episodic-memory condition (see points below the reference line, right graph, Figure 2), there was no significant difference in *k* scores between the episodic-memory condition and control condition, *z* = 1.48, *p* = .138.

#### A fragile effect: statistical approach for *k* determines results

Experiment 1 partially replicated Lempert and colleagues’ (2017) findings. Although results indicated a significant difference in participants’ delay discounting between the episodic-memory and control conditions, the effect of episodic memories on delay discounting was only evident with the AUC measure and not with the *k* measure adopted by Lempert and colleagues. Lempert et al. analysed *k* slightly differently, however, comparing log(*k*) values with a paired-samples *t-*test. We found that log transforming normalised *k* values (but not AUC values). Therefore, we repeated their (log)*k* analysis and still found no significant difference between the episodic-memory and control conditions, *t*(54) = 1.52, *p* = .134. We did, however, repeat their analysis with natural log (ln) scores and found that participants had significantly lower levels of delay discounting in the episodic-memory condition compared with the control condition, *t*(54) = 2.00, *p* = .050, Cohen’s *d* = 0.46 (the same effect size as Lempert et al). Therefore, perhaps the effect of episodic memory on delay discounting is fragile, such that it may depend on the statistical approach researchers use.

#### AUC is perhaps more likely to capture effects of episodic memory than *k*

In contrast to *k*, AUC might be more likely to capture effects of episodic memory on delay discounting. Meta-analyses by Olsen (2020) and Ye et al. (2022) found that the effect of EFT on delay discounting is larger when using AUC compared with *k.* In Experiment 1, one contributor to this might be the two-step process we used to estimate *k* (following Lempert et al., 2017). To estimate *k* required fitting two models: one to estimate the indifference points (also used to calculate AUC) and another to fit the hyperbolic model. This might have introduced more noise into the *k* measure than AUC. Perhaps as a result, AUC values were less variable in Experiment 1. Indeed, the IQRs for *k* were larger than for AUC (see Table 2). Where variability is higher, differences between conditions are less likely to reach significance. Another possible reason that we find an effect with AUC and not *k*, is that *k* does not capture indifference points that deviate from the hyperbolic model, and these deviations might reflect effects of episodic memory. AUC can capture these experimental effects because it directly tracks individuals’ indifference points. To illustrate, there were four participants for whom AUC scores indicated lower levels of delay discounting in the episodic-memory condition while their *k* scores indicated higher levels of delay discounting in the episodic-memory condition. These participants’ *R*^2^ values for the hyperbolic model (*k*) in the episodic-memory condition were < 0.4 and well below the median and thus indicated a poor fit. AUC may provide a more accurate summary of the pattern of indifference points than *k* and therefore be more sensitive to finding small and fragile effects.

#### Are memory effects on delay discounting due to episodic thinking?

Despite the different findings that emerged between AUC and *k*, the results overall indicated that episodic memory decreased delay discounting. A critical question that arises is whether this effect was due to the episodic nature of memories (i.e., memory of a *specific* event). This is because the effect of EFT, which is reliable and strong (Rung & Madden, 2018), may be due to this type of thinking being episodic (i.e., specific future events; Palombo et al., 2016). EFT and episodic memory share a similar cognitive process (Addis, 2018; Schacter & Addis, 2007), such that both draw on episodic thinking and simulate events in the mind, therefore episodic memory may also need to be specific events to decrease delay discounting.

The episodic thinking component of EFT has been investigated by experimentally testing semantic future thinking (SFT, i.e., semantic facts about the future; Chiou & Wu, 2017; Palombo et al., 2016; Wu et al., 2017). This is because SFT is future orientated like EFT but does not have the episodic component. Results can vary depending on the SFT cue, however, such that when the cue is concrete, about money, and is personally relevant, there is an effect of SFT on delay discounting (e.g., *what could you buy for $42 in 4* *months?* Palombo et al., 2016); whereas when SFT cues ask for more abstract facts, like generating benefits for stopping smoking (Chiou & Wu, 2017) or describing your ideal future self (Wu et al., 2017), there is no effect of SFT on delay discounting. Thus, these findings suggest that future thinking may indeed need to be episodic (or more concrete) to decrease delay discounting (also see Experiment 3). Therefore, considering the similarities between EFT and episodic memories (Schacter & Addis, 2007), Experiment 2 tested whether the significant effect found in Experiment 1 was because memories were episodic (i.e., specific events).

## Experiment 2: Do episodic memories have a larger effect on delay discounting than general memories?

Experiment 2 tested whether the effect of episodic memory on delay discounting (as seen in Lempert et al., 2017 and Experiment 1) was due to memory being episodic (i.e., specific events, e.g., last Tuesday, I walked up the hill to university) or also occurred when the memory was general (i.e., categoric events, e.g., the times I walk to university; Conway & Pleydell-Pearce, 2000; Tulving, 2002). While neuropsychological and autobiographical memory research suggest that episodic and general memory are related and may influence each other, they are still conceptualised as two distinct forms of memory (Conway & Pleydell-Pearce, 2000; Greenberg & Verfaellie, 2010; Williams et al., 2007). This raises the possibility that episodic and general memory have different effects on delay discounting. Indeed, given the substantial findings that EFT influences delay discounting (Rung & Madden, 2018), as well as the similarity between prospection and retrospection (Addis, 2018; Schacter & Addis, 2007), and that more concrete and specific SFT decreases delay discounting (Palombo et al., 2016), we hypothesised that participants would have significantly lower levels of delay discounting after the episodic-memory condition compared with after the general-memory condition and control condition. To ensure participants thought and wrote about an episodic or general memory in the assigned condition, however, we also conducted a manipulation check (see below). Also, for Experiment 2, we largely followed Lempert and colleagues’ (2017) procedure again, with methodological changes discussed below.

### Method

#### Participants

Eighty-eight undergraduate students participated in partial fulfilment of a course requirement. We did not collect any demographic information. Two participants were excluded from the final analyses because the researcher supervising the session noticed they became distracted during the study (i.e., looking around the room and interacting with each other rather than focusing on the task and did not respond to a polite request to focus on the experiment). Eighteen participants (21%) displayed unsystematic delay discounting in the control condition, as set out in Experiment 1. This percentage of excluded participants resembles the mean across the delay discounting literature identified by a meta-analysis by K. R. Smith et al. (2018). Therefore, data for 68 participants were used for the analyses. See Table 3 for rates of unsystematic discounting across conditions.

**Table 3.** Experiment 2: Number of participants who met exclusion criteria in the control and -memory conditions.

Control condition only (excluded)	Memory condition(s) only (retained)			Memory condition(s) plus control condition (excluded)		
	Episodic	General	Both	Episodic	General	All three
6 (7%)	6 (7%)	9 (10%)	1 (1%)	5 (6%)	2 (2%)	5 (6%)

#### Procedure

Experiment 2 was similar to Lempert et al., 2017 (and thus Experiment 1), but a general-memory condition was added, the order of blocks differed, and there was no 3-day delay between participants recalling and writing about their memories and making their delay-amount trade-off choices. Episodic memory cues were the same as in Experiment 1 (e.g., *A time I went to a museum*), whereas the general memory cues started with *The times (e.g., The times I went to a museum*). In addition, to ensure participants recalled episodic and general memories, instructions were adapted using the Autobiographical Memory Test (AMT; Williams & Broadbent, 1986) and the Autobiographical Memory Test Revised (AMT-R; Dalgleish et al., 2007), which are typically used to measure memory specificity (AMT) and general memories (AMT-R). That is, for the episodic-memory condition, an episodic memory (i.e., specific memory) was clearly defined as an event that happened at a particular time and place that lasted less than a day, along with an example (i.e., 2 weeks ago, I ate lunch with my friend at the waterfront). For the general-memory condition, the instructions emphasised that participants recall a category of events (i.e., a series of similar events that have happened at different times) and an example was also provided (i.e., I always enjoy eating lunch with my friend at the waterfront; see https://osf.io/62gxb/?view_only=for full instructions).

**Figure 3.**
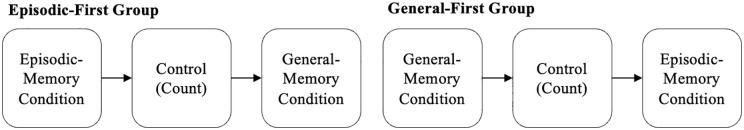
Experiment 2: Order conditions.

Participants were randomly assigned to one of two order conditions (see Figure 3). In the episodic-first group, the participants began with the episodic-memory condition, then completed the control condition, and finished with the general-memory condition. The general-first group completed the experiment in the reverse order. The control condition was placed between the two memory conditions for all participants to reduce the likelihood of carryover effects across the two crucial conditions. Conditions did not alternate across blocks like in Experiment 1 and Lempert et al. (2017) because this might have made it more difficult for participants to keep track of when to recall episodic versus general memories. Condition order was therefore added as an independent variable in the analyses, because research suggests that delay discounting decreases as participants make repeated delay-amount trade-off choices (Neff & Macaskill, 2021; Olsen et al., 2018). Effects on delay discounting are therefore more likely to be observed when the manipulation (e.g., EFT) is presented later in the session, and thus produce order effects (also see Yi et al., 2017).

In the episodic- and general-memory conditions, we asked participants to rate each memory (on a 5-point Likert-type Scale) on its vividness, importance, valence, and the extent to which the memory taught them something about their life. These scales slightly differed from the procedure used by Lempert and colleagues (2017) to answer another research question not discussed here. In the control condition, we asked participants to count in their heads instead of relaxing as in Lempert and colleagues’ study, to ensure they did not recruit episodic or general memories, but completed the same bored, tired, and good Likert-type scales described in Experiment 1.

Other methodological differences from Lempert and colleagues’ (2017) study (as well as Experiment 1) were that participants wrote about five memories per memory condition (instead of 10) and completed the study in one session (instead of two). The delay discounting choices also differed, such that the larger, later monetary reward remained constant at NZD$100. This meant that only one model fitting step was needed to estimate *k*, perhaps reducing noise in the measure. The larger, later amount of NZD$100 was paired with the delays of 6, 30, 60, 100, and 180 days; whereas the smaller, sooner amounts (NZD$15, NZD$30, NZD$45, NZD$60, NZD$75, NZD$90) were always received “now.” Every combination of delay and amount was presented in random order. Indifference points were also calculated differently due to holding the larger, later constant as explained above. Indifference points were therefore defined as the midpoint between the lowest smaller, sooner amount chosen for that delay and the next-smallest amount offered. Where a participant never chose the smaller, sooner reward, we set the indifference point for that delay at NZD$100.

##### Manipulation check

A manipulation check was conducted to test whether participants wrote about the type of memory they were instructed to for the condition (guided by Dalgleish et al., 2007; Williams & Broadbent, 1986; Williams et al., 2007). Two coders, who were unaware of the condition, independently rated each memory as general or episodic, and then cross-referenced their ratings with each other. The two coders came to a 99% consensus and resolved disagreement through consensus: 91% of memories in the general condition, and 90% of memories in the episodic condition, were of the type instructed. All participants were included in the analyses presented below, however, as excluding data from participants who did not follow instructions for one or more memories did not change the results presented.

### Results and discussion

#### Descriptive statistics, normality testing, and statistical analyses plan

**Figure 4.**
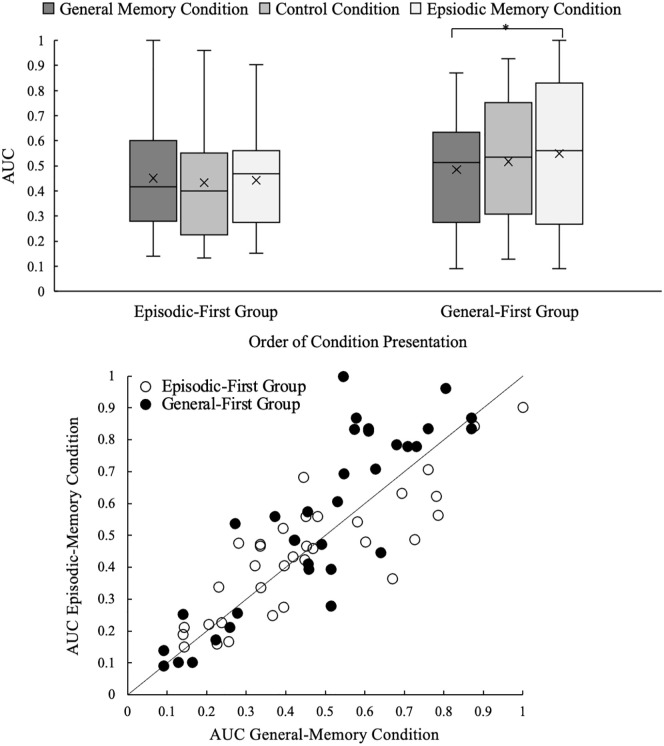
Experiment 2: Distributions of AUC by condition. Note. Top graph: boxes are interquartile range, *X*s are means, and lines are range minus outliers. * indicates *p* < .05. Bottom graph: points above the *x* = *y* reference line indicates participants with lower levels of delay discounting in the episodic-memory condition.

The median and IQR for memory valence suggests that participants recalled positive memories and followed the task instructions (see Table 4). Consistent with visual analysis of Q-Q plots, Shapiro–Wilk tests indicated *k* distributions were significantly non-normal in the general-memory (*W* = 0.32, *p* < .001), episodic-memory condition (*W* = 0.45, *p* < .001), and control (*W* = 0.65, *p* < .001) condition. Log transformations failed to normalise *k* values in all conditions. Therefore, a robust ANOVA was conducted in R using the WRS2 package (see Mair & Wilcox, 2020) to assess the effects of condition and order. A robust ANOVA tests for differences using trimmed means—we used a 20% trim as recommended by Mair and Wilcox. A robust ANOVA was used because there is no non-parametric test that can detect an interaction between two independent variables. AUC values were normally distributed in the general-memory (*W* = 0.96, *p* = .220) and control (*W* = 0.94, *p* = .070) conditions. The episodic-memory condition was marginally non-normal (*W* = 0.93, *p* = .030), but examination of the Q-Q plots suggested deviations from normality were minor (see Figure 4 for distributions). Therefore, a standard ANOVA was used to assess the effects of condition and order for AUC.

**Table 4.** Experiment 2: Descriptive statistics.

Measure	Episodic-memory condition		General-memory condition		Control condition	
	Median	IQR	Median	IQR	Median	IQR
*k*	0.015	0.005–0.036	0.016	0.007–0.044	0.016	0.008 - 0.056
*R* ^2^	0.72	0.46–0.84	0.76	0.43–0.88	0.80	0.63 - 0.88
Valence (i.e., positivity)	4.50	4.20–4.80	4.40	4.20–4.60		
Importance	3.60	3.20–4.10	3.80	3.40–4.20		
Meaning	3.00	2.50–3.50	3.60	3.00–4.30		
Vivid	4.00	3.60–4.50	3.80	3.60–4.40		
Bored					3.90	3.50 - 4.40
Tired					4.20	3.90 - 5.00
Good					3.00	1.90 - 3.20
Relaxed					3.20	2.40 - 4.00

*Note. N* = 68. *R*^2^ values could not be calculated for three participants in the general-memory condition and two in the control condition because their indifference points were identical for every delay. Scales ranged from 1 to 5, with higher scores indicating higher levels of that factor. See Figure 4 for distributions of AUC values.

#### Key finding: no reliable effect of episodic memory on delay discounting

For *k*, a robust 3(condition: general, episodic, control) × 2(order: general-first, episodic-first) robust ANOVA indicated no significant effect of condition, *F*(2, 30.90) = 0.49, *p* = .612. There was also no effect of order, *F*(1, 37.51) = 0.35, *p* = .560; and no order by condition interaction, *F*(2, 30.90) = 0.38, *p* = .690. For AUC, an ANOVA indicated no significant effect of condition, *F*(2, 132) = 2.03, *p* = .135, η²p = .03; or order, *F*(1,66) = 1.93, *p* = .169, η²p = .03; but did indicate a significant order by condition interaction, *F*(2, 132) = 3.29, *p* = .040, η²*p* = .05. A follow up one-way ANOVA (condition 3 levels: general, episodic, control) indicated that AUC differed across conditions when participants completed the general-memory condition first, *F*(2, 64) = 4.60, *p* = .014, η²p = .13; but not when participants completed the episodic-memory condition first, *F*(2, 68) = 0.43, *p* = .650, η²p = .01. Furthermore, 19 of 33 (58%) participants in the general-first group demonstrated lower levels of delay discounting in the episodic-memory condition compared with the general-memory condition, whereas in the episodic-first group, 17 of 35 (49%) participants showed this effect. Indeed, follow up *t-*tests revealed that participants in the general-first group showed significantly lower levels of delay discounting in the episodic-memory condition than the general-memory condition, *t*(32) = 2.55, *p* = .016, Cohen’s *d* = 0.44. The general-first group AUC scores in the general, *t*(32) = 1.63, *p* = .110, Cohen’s *d* = 0.28, and episodic-memory conditions, *t*(32) = 1.80, *p* = .082, Cohen’s *d* = 0.31, did not significantly differ from their AUC score in the control condition, however. In sum, episodic memories decreased participants’ delay discounting in comparison to general memories (but not in comparison to the control), and only when they were recalled last.

One explanation for this order effect is that delay discounting was reduced both later in the session and when participants were cued to think about episodic memories. That is, participants seem to decrease their delay discounting as they progress through an experiment and make repeated delay-amount trade-off decisions (Neff & Macaskill, 2021; Olsen et al., 2018) and episodic memories may help to decrease delay discounting (e.g., Experiment 1; Lempert et al., 2017). Thus, these two factors might have amplified each other in the general-first group (as they completed the episodic-memory condition last) and counteracted each other in the reverse order. The conclusion that these conditions differed meaningfully is, however, tempered by the fact that neither differed significantly from the control condition.

Thus, the results of Experiment 2 suggest that the effect of episodic memories on delay discounting is fragile. Lempert and colleagues (2017) and Ciaramelli and colleagues (2019) found episodic memories reduced delay discounting, as did we in Experiment 1, but the non-significant findings of Experiment 2 and those of Lempert et al. (2020) suggest that whether researchers observe an experimental effect of episodic memories on delay discounting perhaps depends on specific factors. It also suggests that episodic thinking is perhaps most beneficial for decreasing delay discounting when it is future orientated.

## Experiment 3: Are episodic and future thinking key components to decreasing delay discounting?

Experiment 3 therefore investigated whether EFT has a larger effect on delay discounting than episodic memories and, if so, whether this was because EFT is both episodic *and* future orientated. For Experiment 3, we therefore compared delay discounting with EFT, SFT, episodic memory, and “no instruction” cues. Comparing episodic memory to EFT isolated the future orientation component and comparing EFT to SFT isolated the episodic thinking component. While researchers are starting to compare the components of EFT and the effect it has on delay discounting (Rung & Madden, 2018), to our knowledge no studies have compared all these conditions with each other and with a “no instruction” task. Experiment 3 therefore had a “no instruction” (i.e., participants only completed the delay discounting task) control task. The “no instruction” control provided a delay discounting baseline and allowed us to compare the relative importance of episodic and future thinking components by comparing episodic memories, SFT, and the “no instruction” conditions with each other. In addition, to further investigate changes in delay discounting across the session (Neff & Macaskill, 2021), participants completed the “no instruction” control at both the beginning and end of the experiment.

For the SFT condition, we adopted an SFT manipulation that has previously decreased delay discounting (i.e., we asked participants to identify items that a person could purchase with NZD$1,000 in the future; Palombo et al., 2016). We also selected an SFT manipulation that was most comparable to the EFT and episodic-memory conditions, such that all manipulations overlapped with the content of the delay discounting task. That is, the amount of money in the SFT condition matched the larger, delayed amount in the discounting task (NZD$1,000), and the EFT and episodic-memory condition delays (i.e., imagine a future/recall a past event at “x” delay) matched the delays in the delay discounting task (i.e., 0–7 days, about 2 weeks, about 1, 3–4 , and 6–8 months, and 1 year). To discourage participants from imagining future scenes or episodic memories for the SFT task, participants in the SFT condition were also asked to describe the item (e.g., what does it look like? Where could you buy it from?) and were not provided specific future delays and instead asked about items in the future more generally.

In addition, several procedural features were also included to enhance any effect of the three manipulations on delay discounting. For instance, cues for the events and items were placed directly above the delay-amount trade-off choices rather than on the screen beforehand (like in Experiments 1 and 2, and Lempert et al., 2017) to promote EFT/SFT/episodic memory during the delay discounting task. Participants were asked not to make their decision based on their cue, but rather to imagine the item/event vividly before making their choice. Participants also generated their own events, instead of selecting from a list of cues as in Lempert et al., Experiments 1 and 2. This approach allowed participants to select events that were highly vivid and positive for them personally.

Experiment 3 also tested for demand characteristics and put measures in place to manage potential participant fatigue. Although research suggests that the effect of EFT is not wholly explained by demand characteristics (Benoit et al., 2011; O’Donnell et al., 2019), an overlap in content between the SFT, EFT, episodic memory tasks, and the delay discounting task could inflate demand characteristics. Experiment 3 therefore also evaluated whether participants were able to discern the experimental hypotheses. To reduce participant fatigue, a titrating amount procedure (Mazur, 1987) was also used, instead of the fixed choice trials used in Experiments 1 and 2 (and Lempert et al., 2017). A titrating procedure reduced the number of choices a participant had to make (see “Method” section for more detail). To also help with potential fatigue, the experiment was conducted over two 1-hr sessions that were a week apart. Participants were also not given any time limit to write about their item and event. In addition, unlike in Experiment 1 and the study by Lempert et al., but as in Experiment 2, there was no delay between writing about their items/events and the delay discounting task.

The main hypothesis for Experiment 3 was that participants would have lower levels of delay discounting in the EFT condition compared with the “no instruction,” episodic-memory and SFT conditions, consistent with previous research (e.g., Benoit et al., 2011; Chiou & Wu, 2017; Dassen et al., 2016; O’Donnell et al., 2017; Stein et al., 2016; Wu et al., 2017). We also hypothesised that participants would have lower levels of delay discounting in the episodic-memory and SFT conditions compared with the control condition (Lempert et al., 2017; Palombo et al., 2016). This latter hypothesis was made tentatively, considering the mixed and limited findings of episodic memory and SFT on delay discounting.

### Method

#### Participants

Participants were 96 undergraduate students who participated in partial fulfilment of a course requirement. We did not collect any demographic information. Four participants did not follow instructions, so their data were removed. The remaining 91 participants were examined for unsystematic discounting. An additional five participants (6%) demonstrated unsystematic discounting at control Time 1 (see procedure below for detail) so their data were removed (see Table 5 for levels of unsystematic discounting in other conditions for reference). Therefore, analyses included data from 87 participants.

**Table 5.** Percentage of participants with unsystematic discounting by condition.

	Control Time 1	EFT	Episodic memory	EPT	Control Time 2
Experimental condition + control Time 1 (excluded)	5 (6%)	2 (2%)	3 (3%)	2 (2%)	3 (3%)
Experimental condition only (retained)	–	34 (39%)	13 (15%)	15 (17 %)	6 (7%)

Note. Some participants appear multiple times across table cells due to unsystematic discounting in multiple conditions, thus top row adds to more than total exclusions.

#### Procedure

Experiment 3 was a within-participant design that included an EFT, SFT, episodic-memory condition, and “no-instruction” control (i.e., no task before the delay discounting task) condition. To reduce participant fatigue, the experiment was conducted over two 1-hr sessions a week apart.

Participants began with the control condition (i.e., control Time 1) and then two out of three manipulation conditions (i.e., EFT, SFT, episodic memory) were selected at random. A week later, they completed the other manipulation condition that they did not complete in the first session and then the control condition again (i.e., control Time 2). Participants were asked to pick and write about six vivid, positive, personally relevant, and exciting future and past events at different time frames (one for each delay range; 1–7 days, about 2 weeks, about 1, 3–4, and 6–8 months, and 1 year). We provided a range to increase the likelihood that participants could identify events they had planned or could recall. For the SFT, they were asked to identify items that a person could purchase with NZD$1,000 in the future (like in Palombo et al., 2016). They were also asked to write their own cue that corresponded to their future imagining, episodic memory, and item. For the EFT and episodic-memory conditions, participants were also asked to select a date in a calendar of when the event could or did occur.

After writing about their events or items, participants immediately completed the delay discounting task. We used a titrating amount procedure (Mazur, 1987) instead of the fixed choice trials used in Lempert et al. (2017) (and also in Experiments 1 and 2). The titrating amount procedure reduces the number of choices a participant must make, and the effects of episodic memories (Ciaramelli et al., 2019) and EFT (Stein et al., 2016) on delay discounting have been observed using this procedure. In the titrating amount procedure, the size of the smaller, sooner reward is adjusted following each choice. The smaller, sooner amount began at NZD$500, and the participant chose between this amount and NZD$1,000 after a specific delay (i.e., a delay randomly generated between the following ranges: 1–7 days, about 2 weeks (11–17 days), about 1 month (21–35 days), 3–4 months (84–126 days), 6–8months (168–245 days), and 1 year (336–391 days). That is, we selected a delay within the range participants were asked to generate events for in the EFT and episodic-memory conditions. Delays were generated identically in all conditions.

If the participant chose the smaller, sooner reward, the size of the smaller, sooner reward was decreased by NZD$50 for the next trial at that delay. If they chose the larger, later reward, the size of the smaller, sooner reward was increased by NZD$50 for the next trial at that delay. Participants made six choices about each delay. The indifference point for each delay was defined by taking the mean of their final smaller, sooner amount and the next smaller, sooner amount after one additional adjustment.

We also changed how the cues were presented to make the procedure more consistent with the approach typically taken when studying the effect of EFT on delay discounting. Specifically, above each choice, each participant’s (SFT, EFT, or episodic memory) cue for that delay (or the word “choose” for the control) was presented in red and centred above the two choices. Participants were instructed to not base their decision on the cue, but rather vividly imagine the memory, future event or item, and then make their decision.

For each cue, participants rated (on 7-point Likert-type scales) how often the cue made them think about the associated image during the delay discounting task, how vivid and exciting it was, the valence (i.e., positivity), and how expensive the event or item is. In addition, for each cue, participants were asked what picture they had in mind (as in Palombo et al., 2016) to evaluate whether they thought about a scene in the EFT and episodic-memory conditions or an object in the SFT condition. That is, thinking about a scene in the past indicates recalling an episodic memory while thinking about a scene in the future indicates engaging in EFT. There were three options: (1) nothing (i.e., either nothing or only a vague image was pictured), (2) objects but not a scene (i.e., individual items or objects were pictured in isolation but not as part of a scene), or (3) a scene/scenario (i.e., an entire layout was pictured, including objects; the image could be static or dynamic, such as an unfolding scenario). At the end of the experiment, they were asked what they thought the purpose of the study was and what the hypotheses were (as in Stein et al., 2016).

### Results and discussion

#### Descriptive statistics, normality testing, and statistical analyses plan

For Experiment 3, *k* was not used because the median and IQR for *k*’s *R*^2^ values were low in the EFT condition, which suggested the hyperbolic model did not provide a good description of the data. Tests of normality (Shapiro–Wilk and visual inspection of histograms depicting data distributions) showed that AUC was not normally distributed (all *p* < .05, except for the episodic-memory condition *p* = .083), and log transformations failed to normalise these data. Therefore, non-parametric tests were used.

#### Key findings

##### EFT decreases delay discounting, other conditions do not

**Figure 5.**
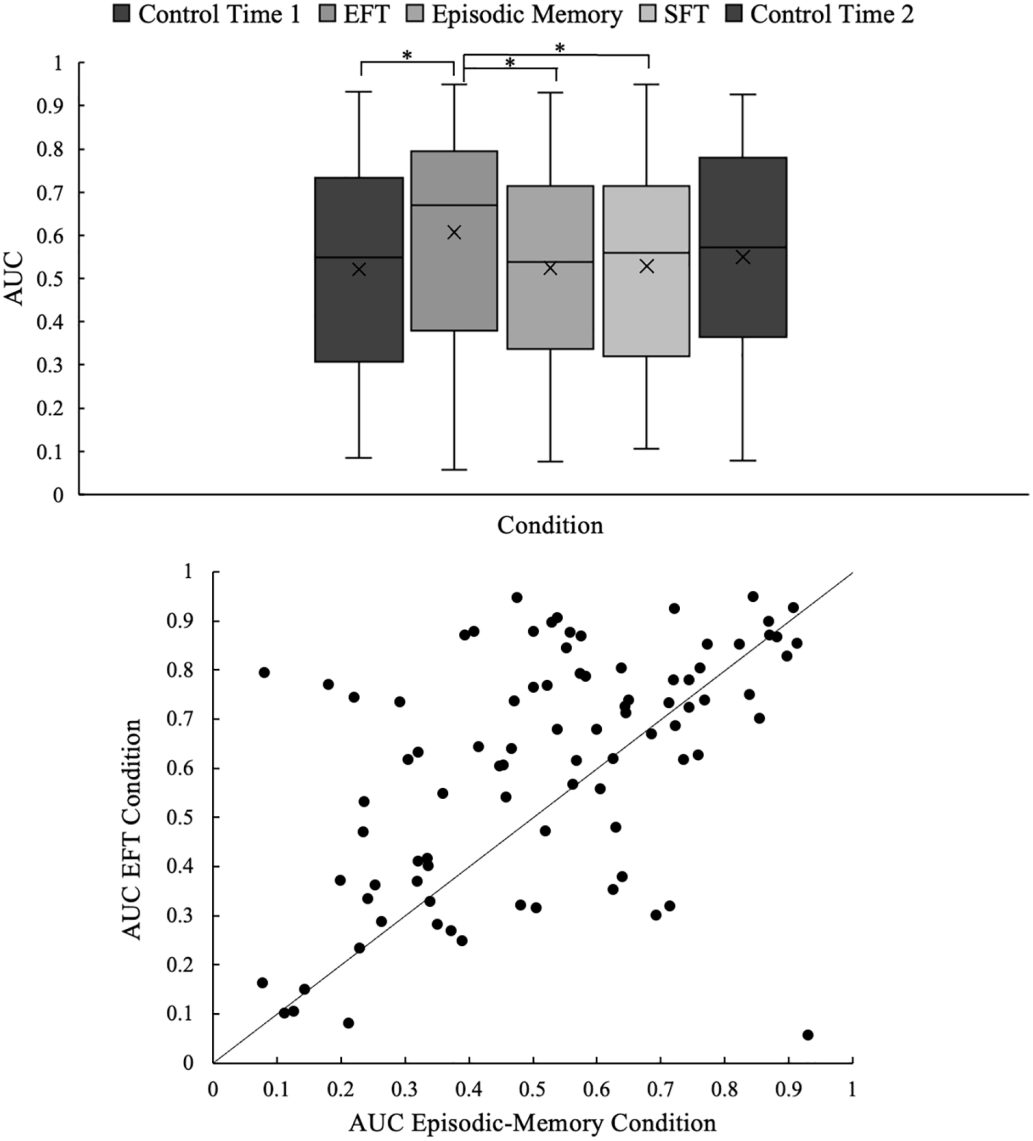
Experiment 3: Distributions of AUC by condition. Note. Top graph: boxes are IQR, lines are range, *X* is mean. * indicates significant difference with Bonferroni-corrected alpha. Bottom graph: points above the *x* = *y* reference line indicates participants with lower levels of delay discounting in the EFT condition compared with the episodic-memory condition, whereas points below the line indicate the opposite. Points on the line indicate no change in delay discounting between conditions.

A Friedman test revealed a significant main effect of condition, *X*^2^(4) = 27.29, *p* < .001. Follow-up Wilcoxon signed-rank tests showed that participants had significantly higher AUCs (i.e., lower levels of delay discounting) in the EFT condition compared with their AUC scores in control Time 1 (*Z* = −3.86, *p* < .001, *r*_rb_ = .48), episodic memory (*Z* = -3.61, *p* < .001, *r*_rb_ = .45) and SFT (*Z* = -3.72, *p* < .001, *r*_rb_ = .46) conditions (see Figure 5 and Table 6). That is, as predicted, EFT significantly decreased delay discounting in comparison to SFT, episodic memories, and the “no instruction” control (Benoit et al., 2011; Chiou & Wu, 2017; Dassen et al., 2016; O’Donnell et al., 2017; Stein et al., 2016; Wu et al., 2017). There was also a difference between control Time 2 and EFT (*p* = .009) but this did not reach significance with Bonferroni-corrected alpha. There was no difference in AUC scores between the episodic-memory, SFT, and control conditions, indicating that thinking needs to be both future orientated and episodic to reduce delay discounting. When analyses were repeated with the full dataset, all significant differences remained (see supplementary material) suggesting that significant differences were not driven by a differential impact of exclusions on the control condition. There was a higher rate of unsystematic discounting in the EFT condition (see Table 5), suggesting that in this condition unsystematic discounting reflected the impact of cues rather than participant inattention or misunderstanding.

**Table 6.** Experiment 3: *p* values from post hoc comparisons using Wilcoxon signed-rank tests.

	EFT	Episodic memory	SFT	Control 2
Control 1	.000*	.454	.896	.011
EFT		.000*	.000*	.009
Episodic memory			.902	.068
SFT				.113

* Significant at Bonferroni-corrected alpha level of .005. Pattern is unchanged using the sequential/Holm Bonferroni method.

##### Episodic memory and control Time 1 condition were statistically equivalent

The non-significant difference between the episodic-memory condition and the control conditions in Experiments 2 and 3 raised the possibility that participants’ AUC scores were equivalent between the episodic-memory and control conditions. We therefore used two one-sided *t-*tests (TOST) to evaluate this possibility. TOST is an equivalence test that allows researchers to support the hypothesis that two means are statistically equivalent if the effect size is within a lower and upper equivalence bounds (guided by Lakens, 2017; Lakens et al., 2018). We set the equivalence bounds to ± a Cohen’s *d* of 0.2 (small effect size).

Participants’ AUC score in the episodic-memory condition was statistically equivalent to their AUC score at control Time 1, lower bound: *t*(86) = 2.01, *p* = .024; upper bound: *t*(86) = 1.72, *p* = .044. Their AUC score in the episodic-memory condition was not statistically equivalent to their AUC score at control Time 2, however, due to a non-significant difference at the lower bound, lower bound *t*(86) = 0.148, *p* = .440; upper bound: *t*(86) = 3.58, *p* < .001. This meant that the effect between these two conditions (episodic memory and control Time 2) was *undetermined.* Note that the means and medians were in the unexpected direction, with lower AUC scores in the episodic-memory condition. Thus, Experiment 3 found no evidence that recalling episodic memories reduces delay discounting. TOST results should be interpreted with caution, however, given non-normal distributions.

##### Control Time 1 versus control Time 2: no significant change

To examine whether delay discounting rates decreased across the session, we repeated the same “no instruction” control condition at the beginning and end of the experiment. The median AUC was higher at the end of the second session (i.e., control Time 2) than at the beginning (i.e., control Time 1), consistent with the order effect observed in Experiment 2, but this increase did not reach significance with a Bonferroni-corrected alpha, *W* = 1,315, *p* = .011 (see Table 6).

##### Manipulation check: only EFT and episodic memory produce mental scenes

**Figure 6.**
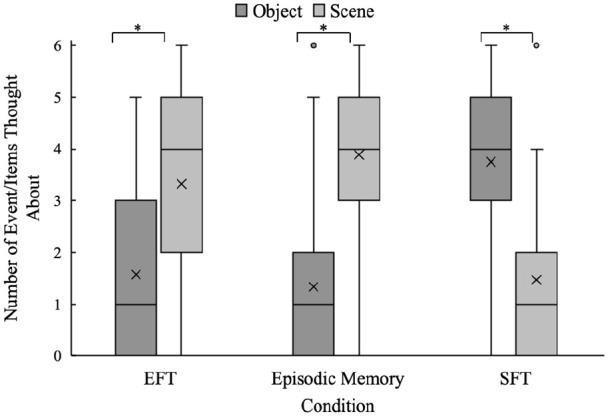
Experiment 3: Distributions of participants’ ratings of the content of their thoughts during conditions. Note: Boxes are IQR, lines are range, *X* is mean. *indicates significant difference at .05 alpha level.

The current results are consistent with the proposal that episodic thinking needs to be future orientated to reliably decrease delay discounting. To test whether this effect was due to episodic thinking, a manipulation check was conducted, and participants were asked whether they thought about scenes or objects for each delay/cue in the EFT, SFT, and episodic-memory conditions (see Figure 6 for distributions). The results revealed that participants thought about objects more than scenes in the SFT condition (*Z* = −6.13, *p* < .001, Bonferroni-corrected alpha level = .007), and scenes more than objects in the episodic memory (*Z* = −6.32, *p* < .001, Bonferroni-corrected alpha level = .007) and EFT (*Z* = −4.81, *p* < .001, Bonferroni-corrected alpha level = .007) conditions as instructed. Thus, participants thought about scenes in both the episodic-memory and EFT conditions, yet only EFT decreased delay discounting. This suggests that episodic thinking needs to be future orientated to have a subsequent effect on delay discounting. In addition, the difference in participants’ delay discounting between the EFT and the other conditions could not be explained by ratings of how vivid, exciting, expensive, or positive the events/items were (see supplementary material for more information). In summary, for the vivid, exciting, expensive, and positive ratings to explain the pattern of effect observed, the EFT condition would need to differ from other conditions on one or more dimensions, but this was not the case.

##### No demand characteristics evident

The results also cannot be explained by demand characteristics. Only 15% of participants identified the predicted effect that EFT would produce less discounting (see Table 7). Another 22% suggested the cues would affect monetary decision making but not in the direction of the predicted effect. Thus, 37% described the possibility that cues would affect choice. This is comparable to the 39% who incorrectly described our predictions and results. Furthermore, demand characteristics would only account for the pattern of results if participants who could identify the hypothesis *also* produced lower levels of delay discounting in the EFT condition. Only 12% of the total sample both identified the hypothesis and decreased delay discounting in the EFT condition, however. Overall, these results show that demand characteristics are an unlikely explanation for the results of the current experiment.

**Table 7.** Experiment 3: Participants’ answers to the questions assessing demand characteristics coded into categories.

Type of coded answer	Percentage of participants
Guessed that the researcher was interested in how people make decisions about money, with no mention of the cues	10% (8/82)
Incorrectly guessed the researcher’s predictions and results	39% (32/82)
Stated that they did not know the researcher’s predictions	1% (1/82)
Correctly guessed the researcher’s predictions, but inconsistent with results (e.g., EFT *and* EPT will reduce discounting)	13% (11/82)
Guessed researcher’s prediction that is consistent with results (i.e., only EFT will reduce discounting)	15% (12/82)
Correctly identified research question, but did not clarify direction of prediction (e.g., investigated whether EFT will affect decisions about money)	22% (18/82)

## General discussion

Our research aimed to test whether recalling episodic memories can decrease delay discounting. As the effect of EFT on delay discounting is well established (Rung & Madden, 2018), and future and past episodic thinking both involve simulating events in the mind (Addis, 2018; Schacter & Addis, 2007), we hypothesised that episodic memories could also be helpful. An additional reason for this hypothesis was that, like EFT, episodic memories may also help one to connect to their future self (Hershfield, 2011; Lempert et al., 2017), as well as divert attention away from the present (Ciaramelli et al., 2019; Smallwood et al., 2013), which may consequently help people to wait for larger rewards.

Only two studies have found an effect of episodic memories on delay discounting, however (i.e., Ciaramelli et al., 2019; Lempert et al., 2017). Of the two, Lempert and colleagues provided direct evidence of positive episodic memory decreasing delay discounting, whereas Ciaramelli and colleagues’ effect was only seen during post hoc analyses. Thus, Lempert and colleagues’ experimental design provided the basis for our research, particularly for Experiments 1 and 2. Only Experiment 1, however, found that positive episodic memories decreased delay discounting compared with a control condition (i.e., relax), while the other two experiments did not. In addition, Experiment 3 found that only EFT decreased delay discounting and not episodic memory or SFT. Our pattern of results suggests that (1) the effect of episodic memory on delay discounting is fragile, such that the effect is small and depends on several factors; and (2) the most reliable effect of episodic thinking on delay discounting is when it is future orientated. That is, if episodic memory had a robust, significant effect on delay discounting, this effect would be present regardless of a range of well-established and sensible data analysis and experimental design choices. Whereas EFT appears to be robust across variations of experimental design and analysis choices (Rung & Madden, 2018).

Therefore, the fragile effect of episodic memory on delay discounting perhaps depends on experimental or contextual factors (e.g., how the delay discounting rate is calculated—see Experiment 1 discussion). Interestingly, Experiment 1 was the most like Lempert and colleagues’ (2017) experiment that also found an effect of positive episodic memories on delay discounting. The most notable experimental similarity between the studies is that there was a 3-day delay between choosing episodic memories and completing the delay discounting task. Although we did not instruct participants to recall their memories over the 3 days, they may have continued to recall their memories over the delay and consequently used it to guide their decisions to choose larger, later rewards (Bluck, 2003; Bluck & Alea, 2002; Daniel et al., 2016; Pillemer, 1992). That is, perhaps the 3-day delay gave participants time to reflect on how they saved or waited for that experience and therefore wanted that experience again. They therefore avoided discounting that experience and thus opted to wait for larger, later amounts during the delay discounting task (see Daniel et al., 2016 for further discussion).

Furthermore, the memories’ vividness may have increased after a 3-day delay as they had time to keep recalling their memories (Bluck et al., 1999; Nadel et al., 2007), and this may have helped to lower discounting (Ciaramelli et al., 2019). In Experiments 2 and 3, delay discounting choices were immediately after remembering, which may have not been enough time for participants to use their memory to guide their delay discounting choices and/or for them to be vivid enough to have an effect. Thus, perhaps the 3-day delay allowed participants to reflect on their experience, increasing vividness and guiding their decision. This would suggest that episodic memories only decrease discounting in day-to-day decision contexts where there is sufficient time to recall and reflect on the memory. Our interpretation of the 3-day delay is limited, however, as the current experiments were not designed to systematically evaluate the effect of including a delay between writing about memories and assessing delay discounting. Future research could systematically vary the delay between writing about memories and assessing delay discounting to assess the impact of this variable.

Two differences between Experiment 1 and Experiments 2 and 3 might have promoted lower rates of delay discounting in the control conditions in the latter two experiments, which may have made it difficult to observe significant effects of the episodic-memory condition. First, in Experiments 2 and 3, the larger, later amount was held constant, which has demonstrated to reduce delay discounting (Johnson & Bickel, 2002). Second, the larger, later amount was larger (NZD$40 in Experiment 1 vs. NZD$100 in Experiment 2 and NZD$1,000 in Experiment 3), which also reduces delay discounting (e.g., Green et al., 1997). In Experiments 2 and 3, however, AUC values were not near the ceiling and so there was a possibility to observe an effect of episodic memory. Indeed, in Experiment 3 we observed an effect of EFT which indicates it is possible to detect a change in delay discounting between the manipulation and control conditions using a constant and larger, later amount. Thus, these procedural differences are unlikely to explain the differences in effect across experiments. Similarly, a recent meta-analysis (Rösch et al., 2022) found that EFT has a smaller effect on delay discounting in studies using within-participant designs (an effect consistent with the order effects we observed in Experiment 2). Thus, we might have observed a larger effect of episodic memory on delay discounting if we used a between participant design. Lempert and colleagues (2017), however, observed an effect of episodic memory using a within-participant design (and so did we in Experiment 1), and EFT also reliably reduces delay discounting using a within-participant design.

In general, in comparison to the well-established effect of EFT on delay discounting under different experimental conditions (Rung & Madden, 2018), our three experiments show that the effect of episodic memory on delay discounting is fragile—the effect is small and depends on specific experimental factors. Instead, episodic thinking that is future orientated has a more reliable effect on delay discounting in the current and previous studies (Rung & Madden, 2018). In Experiment 3, we tested whether this was because EFT is both future orientated *and* episodic. Indeed, EFT significantly decreased delay discounting compared with episodic memories, SFT (i.e., thinking about items a person could buy in the future) and a “no-instruction” baseline condition. Furthermore, episodic memories and SFT had no significant effect on delay discounting. Taken together, our results suggest that delay discounting is most reliably reduced by thinking that is both future orientated *and* episodic. Although under some conditions SFT and episodic memory can decrease delay discounting, shifting attention away from the present to the future (i.e., SFT) *or* simulating any scene in the mind (i.e., episodic memory) does not reliably decrease delay discounting, particularly in comparison to EFT.

A limitation of Experiment 3, however, is that the SFT and EFT conditions differed in how delays were incorporated into the future thinking we asked participants to engage in. Participants in the SFT condition were instructed to think of an item that a person could buy in the future in general, whereas in the EFT condition we asked participants to consider events at specific delays. Therefore, it could be that the SFT condition had no effect on delay discounting because of a reduced future thinking component. Previous research suggests that the EFT effect does not depend on temporal specificity, however. Peters and Büchel (2010) found comparable EFT effects when participants thought about the future more generally (i.e., when participants were instructed to list events that were all “within the next couple of months” and not tied to a specific date) and when participants thought about the future with specific time points as in typical EFT studies. In addition, both Chiou and Wu (2017) and Wu et al. (2017) described the future component of their SFT and EFT conditions in the same way (using one delay of “1 year”), yet only EFT reduced delay discounting in those studies. Thus, it is unlikely that the generality of the future component of the SFT condition accounts for Experiment 3 results.

Why might EFT have a more reliable effect on delay discounting than episodic memories? Simulating specific future events in the mind may increase personal connectedness to the future self in a concrete and detailed way (Lebreton et al., 2013; O’Donnell et al., 2017), which may encourage decisions and behaviour that benefit the future self (Ersner-Hershfield et al., 2009). Research suggests that when people feel connected to their future selves, they are more likely to save (Hershfield et al., 2011), exercise longer, and have better subjective ratings of physical and mental health (Rutchick et al., 2018). EFT may help a person to see and feel connected to their future self, and thus make subsequent decisions that benefit their future self. Lempert and colleagues (2017) posited that perhaps episodic memories effect on delay discounting works in a similar way, by heightening nostalgia and thus connect people to their future selves. This could be a possibility, but perhaps EFT does so more reliably.

In summary, the current set of experiments demonstrate that episodic memory might promote delay discounting under some circumstances, but this is a fragile effect, such that it is small and depends on several contextual factors. This is particularly the case in contrast to the effect of EFT on delay discounting, which is larger and highly replicable. The fact that the effects of episodic memory and EFT on delay discounting differ in size and reliability suggests that episodic thinking is not enough to decrease delay discounting. Furthermore, although episodic memory and EFT share a cognitive process of simulating events in the mind, our results suggest that past and future thinking may have different functions and effects on behaviour and decision making. Episodic memory may decrease delay discounting under some circumstances, but our findings suggest that the most reliable and stronger effect is when thinking is both episodic *and* future orientated.

## Notes

1. Note that other variables were preregistered for a secondary analysis but are not discussed in this article as they were for other research questions. Experiments 2 and 3 were not preregistered, but data are available on the OSF link.
